# Embodied Interaffectivity in the Emergence and Maintenance of Group Cohesion

**DOI:** 10.3389/fpsyg.2022.822072

**Published:** 2022-07-01

**Authors:** Cheryl Jones, Deborah Pino-Pasternak, Simone Volet

**Affiliations:** ^1^College of Science, Health, Engineering and Education, Murdoch University, Perth, WA, Australia; ^2^Faculty of Education, University of Canberra, Canberra, ACT, Australia

**Keywords:** collaborative learning, embodied interaffectivity, embodied perspectives, group cohesion, interaction analysis, interpersonal affect, socioemotional interaction, teamwork

## Abstract

Group cohesion is an affect-laden construct, with a large body of research indicating its importance for success of teams. Surprisingly, it has received scant attention in collaborative learning contexts, and little is known about its development as dynamically emergent in the spontaneous, interdependent actions of actors during groupwork. This paper details an illustrative case analysis which took an embodied perspective to explore the role of interaffectivity in the emergence and maintenance of cohesion in one small group of university students who reported a highly positive and productive experience of collaborative science activities over a semester. The case analysis made visible group cohesion as unfolding and enactive in the myriad ephemeral and seemingly inconsequential microlevel behaviors that evolved into macro-temporal patterns of positive embodied interaffectivity, magnifying their visibility and collective impact. A fine-grained embodiment lens unveiled how participants cocreated collaborative affordances in actions that involved corporeal orientation as well as use of space, task, and other material artifacts. Task-related humor within routine task interaction offered the potential for establishing group cohesion in early group life, but also posed a potential threat to task-focused cohesiveness, requiring careful modulation at critical task points. Attentiveness not only to the task but importantly, to one another as interpersonal attentiveness, appeared to be a key factor in developing and maintaining group cohesion, also demonstrating collaborative learning as a process of orienting to and understanding tasks *through one another*. An embodiment lens highlighted mutual attentiveness in the ongoing orienter-orientee microprocesses that facilitated group orientation early in group life, and in reorienting to positive embodied interaffectivity when the group reconvened for their joint science activities in subsequent weeks.

## Introduction

Although group cohesion is a much examined construct in organizational psychology (Kozlowski, 2018), there is minimal research on its development in higher education contexts (Thornton et al., 2020). This is despite contemporary demands for teamwork ready graduates (Riebe et al., 2016) who can thrive in workplaces that are increasingly reliant on self-managed teams (Stephens and Lyddy, 2016). Group cohesion can be broadly understood as “group members’ positive attraction to the group” (Kelly and Barsade, 2001, p. 105), often described as a group’s *esprit de corps* (Kelly and Barsade, 2001; Forsyth, 2014), and is widely agreed to be an affect-laden construct (Kozlowski, 2018). Yet, it is only in recent decades that researchers have taken a keen interest in investigating how affect functions “in the development and maintenance of group cohesion” (Van Kleef et al., 2017, p. 159).

Meta-analyses by Castano et al. (2013) and Mathieu et al. (2015) have confirmed the key role of cohesion for group success (i.e., task performance, group member satisfaction). However, despite the plethora of research, little is actually known about group cohesion as a dynamically emergent and affectively charged phenomenon, how it develops in real time, as research has typically relied on cross-sectional data gathered *via* self-report measures. As we are often unaware of our own fleeting everyday behaviors in social encounters, let alone recount them later as potentially relevant (Erickson, 2006; Herrle, 2020), interaction analysis can get to the heart of how collective constructs such as group cohesion actually manifest in dynamic social interaction (Lehmann-Willenbrock and Allen, 2018). Capturing the fine-grained nuances of group interaction is critical for understanding the “in-between” space of intersubjectivity, meaning-making, and social understanding in groupwork contexts. As Bonito and Sanders (2011, p. 344) put it, “When people interact, they progressively provide each other with opportunity spaces that constrain what can and cannot relevantly be said and done.” Such phenomena are integral to our understanding of how interaction inhibits or affords the emergence and evolution of group cohesion.

The widespread use of virtual teamwork in recent years places high emphasis on social interaction when teams actually meet in real time, either by videoconferencing or “in the skin” (Stephens and Lyddy, 2016, p. 14). Given the importance of cohesion for groups to effectively, and autonomously, manage the inevitable challenges of collaboration (Hod and Katz, 2020), we examined how group cohesion emerged and unfolded in one group of university students who reported a highly positive and cohesive interactive experience following their groupwork over one semester. In a review of the collaborative learning literature, Ferreira (2021) proposed that an embodied perspective that highlights the situated corporeality of actors in social interaction, may provide an insightful lens for exploring transversal competencies such as the interpersonal and socioemotional capabilities that are critical for effective collaboration. Therefore, this paper presents a fine-grained case analysis, from an embodied perspective, of the emergence of group cohesion as dynamically evolving (Hendry et al., 2016) and affectively-charged (Knight and Eisenkraft, 2015). This case analysis illustrates the role of what Fuchs (2016) refers to as *embodied interaffectivity*, in the development and maintenance of group cohesion (Van Kleef et al., 2017) in tertiary collaborative learning.

## Conceptual Framework

### Embodied Perspectives of Social Interaction

Embodied perspectives of social interaction have their roots in phenomenology, as described in Merleau-Ponty’s (2012) *Phenomenology of Perception*, which highlighted the way our felt, environmentally situated corporeality through which we experience and make sense of life, was largely overlooked by traditional dualistic mind-body perspectives. Merleau-Ponty refuted a dichotomous perspective of object and conscience. Similarly, embodied cognition theory views our thoughts and actions as not constituted solely by a brain working as an organism separate from its environment but rather as part of a broader internal (corporeal) and external (situation, or environmental) *system*. As such, environmental factors *including* other beings, and the spatial and material also comprise the cognitive system.

Embodied cognition theory is of increasing interest to educational researchers, with a growing corpus of literature in STEM disciplines that have designed embodiment into learning activities, arguing that corporeal involvement can enhance learning (see Skulmowski and Rey, 2017, for a review). Embodied learning is grounded in the notion that students’ learning is processed and understood through their bodies, as cognition extends beyond individual corporeality to one’s environment, including others as well as spatial, and other material phenomena (Walkington et al., 2019). For example, the 4E perspective conceptualizes cognition as embodied, embedded, enacted, and extended (Newen et al., 2018). To date, however, minimal attention has been paid to embodied perspectives in groupwork contexts (Abdu et al., 2021). Yet, as Flood (2018, p. 152) has argued “without considering embodied action in the material environment, many resources that participants use while working to achieve intersubjectivity are not available for analysis and remain unexplored.”

An embodiment view, according to Roth and Jornet (2013), also establishes “a direct connection between thought and affect” (p. 474) thus bridging traditional notions of a cognitive-affect divide. Stapleton (2013) also demonstrated this point by drawing on neuroscientific research to argue that there is now sufficient evidence to suggest the mechanistic interdependence of affect and cognition. As Roth and Jornet (2013, p. 474) put it “social aspects give rise to the shared nature of affect,” such as emotion contagion, collective grief, or euphoria, emotionally charged disagreements, and other common experiences of situated affectivity that moment-by-moment shape social life through vocal, facial, postural, and other modes of expression. Thus, social meaning making is facilitated not only verbally but also through continually unfolding non-verbal phenomena that convey messages almost instantaneously: through pursed lips, a tightening jaw, the slightly turned (cold shoulder), a compassionate gaze, placement of objects, implying implicit communicative messages often not easily, or effectively, conveyed by words (Ferreira, 2021).

This paper adopts an embodied perspective to explore the role of interaffectivity in the emergence and maintenance of group cohesion, of one small group of university students. In so doing, the illustrative analysis presented, acknowledges the inherently animated (Sheets-Johnstone, 2009) nature of actors in social interaction, and highlights the way in which “bodily action in general affords or constrains an interaction” (Ferreira, 2021, p. 1467). This embodied view holistically incorporates the group as situated in its environment, therefore considering other material, and spatial features that together contribute to fundamentally shaping perceptions that cocreate collective social understanding.

### Interpersonal Affect and Embodied Interaffectivity

In recent decades, emotion scholars have expanded theoretical perspectives of affect, integrating its implicitly social, dynamic, and situated nature (Kuppens, 2015), as affect both arises from, and also shapes, social encounters (Mesquita and Boiger, 2014). For example, a sociodynamic model of emotions posits that:

The point is not that emotions occur in response to social events; rather, it is that social interaction and emotions form one system of which the parts cannot be separated… cannot be reduced to each individual’s emotions; nor can the emotions be fully disentangled from the interaction…Moreover, interactions at any point in time are afforded and constrained…Thus, emotional interactions are closely tied to the interpersonal contexts in which they take place (Mesquita and Boiger, 2014, p. 298).

An embodied perspective provides the conceptual lens through which the social and dynamic nature of affect as situated, systemic phenomena can be illuminated, tapping the innate sociality of affect (Fuchs, 2016). For example, phenomenologist Krueger (2011) observed the social information that is available in bodily affect expression, which is consistent with emotion scholars Van Kleef et al.’s (2010) theory of *Emotions as Social Information* (EASI) as providing individuals “insight into their own performance level, their inclusionary status in the group, the norms of the group, and the functioning of the group as a whole” (Van Kleef et al., 2017, p. 159).

Fuchs (2016) introduces the concept of *embodied interaffectivity*, which phenomenologically accounts for an embodied process of the potential for interpersonal understanding through “a process of mutual modification of bodily and emotional states” (p. 195) as we perceive one another’s affective expression in a given context. As such, Fuchs (2016) argues, social encounters do not commence from “isolated individuals and their respective inner states, but from a priority of *intercorporeality* and *interaffectivity*” (p. 195), through which intersubjectivity takes place and common meaning can be negotiated (Fuchs and De Jaegher, 2009).

For Fuchs (2016), embodied interaffectivity encompasses the “spatial phenomena that connect the embodied subject and the situation with its affective affordances in a circular interaction,” leading to “the concept of embodied interaffectivity in…face-to-face encounters” involving a “process of bodily resonance,” coordinated interaction and “mutual incorporation” (pp. 195–196). Fuchs (2016) explains that such processes are grounded in the “intercorporeal memory or implicit relational knowledge that is acquired in early childhood” (p. 196) and while they can also involve higher order cognitive processes such as perspective-taking, “intercorporeality and interaffectivity remain the basis of social cognition” (p. 196).

### Conceptualizing Embodied Interaffectivity in the Emergence and Maintenance of Group Cohesion

An embodiment lens through which to explore group cohesion, aligns with Sheets-Johnstone’s (2009) fundamental view of affect as movement, toward or away from, as attraction to the group through Fuchs (2016) concept of embodied interaffectivity, to illuminate how cohesiveness emerges and evolves over time through the micro-temporal moves of interactivity. As described in the previous sections, an embodiment lens makes visible a systemic perspective of social interaction, what Krueger (2011) calls the “we-space,” which aptly depicts the joint space of groupwork as encompassing the intercorporeality and interaffectivity that Fuchs (2016) conceptualizes. Within this we-space, processes of interaffectivity, which Mühlhoff (2015) describes as the *affective resonance* of jointly created interpersonal phenomena, arises and continually unfolds. In this way, affect is part of the group’s ecology (de Freitas et al., 2019). Embodied views that conceptualize affect as innately collective, social and dynamic phenomena enable group cohesion to be made visible as emergent in the moment-to-moment interdependent actions of participants, a systemic phenomenon of the group’s ecological, we-space. The we-space incorporates not only one another but also interrelations with, and within the situated materiality of the group environment, and the affordances, or constraints therein for cohesiveness to manifest. In this way, an embodied perspective illuminates the processes of Forsyth’s (2014) definition of *group cohesion* as arising from the development of “mutual interpersonal bonds among members and group-level forces that unify the group, such as shared commitment to group goals and esprit de corps” (p. 10).

Despite wide acknowledgment that cohesion is related to effective group functioning “there is little direct empirical research on factors that shape” how it develops and emerges (Kozlowski and Chao, 2012, p. 346). Described below in section “Analytical Focus,” the function of positive embodied interaffectivity in group cohesion was explored through the situated microprocesses of affective co-orientation of the case group interactants. As De Jaegher and Di Paolo (2007) put it, through the microprocesses of interaction, “participatory sense making” can be understood as “the growth of an adaptive system” (p. 496). This is made visible in “patterns of coordination” that “can directly influence the continuing disposition of the individuals involved to sustain or modify their encounter” through actions that “can have the consequence of steering the encounter or facilitating (or not) its continuation” (p. 492).

Although the affective nature of group cohesion has been widely acknowledged, research continues to rely heavily on individual-level self-report measures (Forsyth, 2021) with little known about the real time dynamic and interactive characteristics of its development. Our case group provided an opportunity through which we shed light on this little known phenomenon. The following section describes the context of the case group used to investigate and illustrate an embodied perspective of interaffectivity and its function in the emergence and maintenance of group cohesion.

## Materials and Methods

### Video-Recorded Observations

The case group comprised teacher education students who worked together in nine instances during a first-year university introductory science unit. The six videorecorded episodes presented and analyzed in this paper were from the group’s first two classes working together early in the semester, and one from their final groupwork class late in the semester. The group’s early meetings were selected for analysis given that a large body of organizational research suggests that “cohesion and coordination are most critical to focus on early during the developmental process of teams” as they tend to reach an equilibrium by later phases (Braun et al., 2020, p. 21). This is consistent with seminal literature on group development (Kozlowski and Chao, 2012) and research showing that affective experiences during the initial phases of groupwork influence how groups function in subsequent interactions (Barsade and Knight, 2015). It also reflects research on student groups, such as that of Hommes et al. (2014), in which students themselves relayed the importance for social cohesion to develop early in group life.

### The Case Group

The target group included two females and two males who had not worked together previously. The two males knew of one another slightly from attending a unit in the previous semester (first semester of the course), but none of the members were friends prior to their groupwork. The group’s first activity together was in the second week of the science unit, which is referred to in the Findings section (e.g., “Task and Relational Humor”), as their first lab together. The group was heterogenous in terms of their age: the two females were relatively recent school leavers (under 20-years) and the two males were mature-age (one mid-twenties and one >30-years). Approval for the video-recordings and interviews was granted by the university and undertaken in line with the national research code of conduct for human research, and the students provided their written consent for the video-recordings and interviews.

In an interview conducted at semester end, the group reported highly positive group dynamics, and a cohesive environment in terms of both their relational and task interaction, and therefore was considered highly suitable to explore an embodied perspective of group cohesion. For example, in their interview, repeated reference was made to how well they worked and got along together, and that they were aware that others in their cohort did not have such a positive group experience. Moreover, initial viewing of video-recordings depicting this group *in situ* showed a clear contrast between them and other groups in the same class, as they often stood together around their activities to work, rather than working seated.

### Analytical Focus

An embodied lens magnifies the fine-grained temporality of messages that are continually communicated not only verbally but in myriad ongoing moment-to-moment actions that cocreate what Krueger (2011) called the “we space,” the in-between space of “I” and “other” in interactivity. The case analysis illustrates how this group cocreated a positive and cohesive “we space” in the finely time-scaled and ubiquitous orientation processes of social interaction during their first two groupwork labs together. In ongoing orientation processes that are continuously present in, and fundamental to coordinating social interaction, actors switch between orienter and orientee processes. For example, packing one’s belongings (orienter) with the intention of orienting other/s toward ending a meeting, which orientees may comply with by following suit. However, the orientee is also sense-making, and through participation may in turn adjust the orienter’s perspective. In the case of the orienter moving to close a meeting, the orientee may make a gesture indicating the orienter to wait (which could be as minimal as a slight frown toward the packing actions), orienting the orienter in turn that it is not yet time to leave. The leaving orienter may then adjust their actions, for example ceasing packing and returning attention to the meeting, orienting to the orientee in turn, or alternatively continuing packing (see Stevanovic and Monzoni, 2016, for a fascinating embodied perspective of orienting processes in just such a situation, or De León, 2017, for orienting in a different context). The microprocesses of orienting in social interaction are ubiquitous and often fleeting, for example a wryly arched eyebrow used to attune orientee/s to the orienter’s perspective in the moment, which the orientee may co-orient to with nothing more than a slight widening of the eyes, or a returned eyebrow raise.

In these kinds of small moves, De Jaegher and Di Paolo (2007) describe how, “shift[s] in meaning” can be cocreated through subtle adjustments of one another’s perspectives as an ongoing process which demonstrates the way in which “individual sense-making activities become adjusted through the situation and how a shift in meaning is…created by the interaction dynamics and not just the individuals” (p. 498). Accurately perceiving the intonation of one another’s sense-making enables modulation toward mutual orientation. This sense-making, argue De Jaegher and Di Paolo (2007), is “intentional and expressive; it is essentially embodied in action” (p. 497). For example, Stevanovic and Monzoni (2016) used different social encounters to highlight the key role of actions, including manipulation of material objects, in orientation processes. Their study found that in situations of discord between what was being said and what was being done, participants oriented to actions rather than speech.

Affectivity orients actors to what is considered valuable, or salient in social situations (Fuchs and Koch, 2014; Van Kleef et al., 2017). Hence, the current analysis focused in particular on orienting actions (i.e., orienter-orientee) during early group life that expressed a positive affectively modulated perspective—of their group task or relationally toward one another. The inherently multimodal nature of embodied interactivity (Norris, 2004) can reveal not only the moment-to-moment mechanisms of coordination such as in processes of orienting to one another, but also the breakdown and repair moves that either contribute to, or inhibit sense-making in collaborative learning contexts (Ferreira, 2021). The group’s enactment of orienting, and repair actions were therefore examined as multimodal. That is, that orienting a positive intonation in attempts to coordinate or repair the group’s interaction may be communicated in bodily actions such as gestures, mannerisms, posture, movement toward or away from, manipulation of materials, space, and utterances (Stevanovic and Monzoni, 2016).

Qualitative microanalysis of the video-recordings was informed by Herrle’s (2020) microethnography methodology, and Rödel’s (2020) phenomenological approach to video analysis, which highlights the importance of focusing on participants’ perspectives of the meanings of actions by tracing their attentional focus. That is, rather than attempting to catalog all the potential semiotic resources available in a multimodal analysis, the analyst is instead guided by the actions and interactivity of the participants as they temporally unfold (Goodwin, 2000). Videos of the group’s first two activities were thus viewed in full, first with sound and full transcriptions, which were annotated with notes about actions, including space and materials utilization, corporeal positioning and eye-gaze direction, considered salient for further exploration.

Instances of orientation actions as described above that had a visible positive valence were noted for investigation. Episodes (i.e., multiple participant actions or verbal interactions) that involved highly animated group participation (i.e., all-group laughter and task focus), and turns or episodes involving potential breakdown points (i.e., negative affect expressions or group member/s non-participation) were also noted at this stage. Then, the group’s final lab together was also viewed in full for a perspective of how they interacted over time near the end of semester, following the same process as for the first two labs. Following these steps, the annotated transcriptions of the first two labs at the start of semester and the final lab late semester guided the exploration of whole group positive interaffectivity in the video-recordings.

Once the illustrative excerpts were selected, they were then viewed both with and without sound multiple times in an iterative process to identify the multimodality of salient actions, viewing individual-in-group actions as collective interactivity to capture their “enacted ecology” (Erickson, 2017, p. 60), for example, noting others’ actions while one member was speaking. The salience of actions was guided by what the participants themselves oriented to Rödel (2020). The analytical steps described above were primarily undertaken by the first author and then shared and discussed with co-authors at each step, in the iterative process of developing the illustrative episodes and validation of the observations.

## Findings

Manifestations of embodied interaffectivity as multimodal interaction, in the development and maintenance of group cohesion were evident throughout all of the group’s activities. The embodiment lens made visible the critical role of positive interaffectivity. These manifestations are examined in turn as: orienter-orientee actions affording positive interaffectivity in early group life; (re)orientation processes sustaining group cohesion and positive group-level task engagement; and breakdown and repair of positive interaffectivity to maintain group cohesion.

### Orienter-Orientee Actions Affording Positive Interaffectivity in Early Group Life

The role of affect as an inherently interpersonal and dynamic process in the development of group cohesion as positive *embodied interaffectivity*, was manifested in two ways, first, in explicit, readily visible forms of ongoing task and relational humor, and second in myriad far more tacit, micro-sequential actions that continually signaled interpersonal attentiveness and affective orientation.

Overall, the embodiment analytical lens illuminated the process of mutual orientation toward positive interaffectivity, enacted through humor combined with the close joint attentiveness that facilitates mutual orientation. The following two sections detail how these orientation processes, involving task and relational humor, and attentiveness to one another, unfolded in bodily actions that incorporated spatial, task, and other artifacts of the group environment. The four illustrative figures are representative exemplars of early group life interactivity and orienter-orientee actions affording positive interaffectivity.

#### Task and Relational Humor

Two illustrations from the group’s early activities highlight the nature of continually unfolding micro-temporal, multimodal actions involving orientation toward task-focused humor, in bodily moves such as gesturing, smiling, eye-gazes, spatial manipulation, task artifacts, and talk, and how these combined actions developed into a positive and inclusive group atmosphere. The group’s first lab involved learning about designing an investigation, making observations and inferences, measuring, making predictions, and categorizing and recording results, through two activities: preparing “psylli-slime,” and “oobleck” products. Following hands-on activities, the group also discussed and documented their conceptual reasoning guided by lab book questions.

**FIGURE 1:**
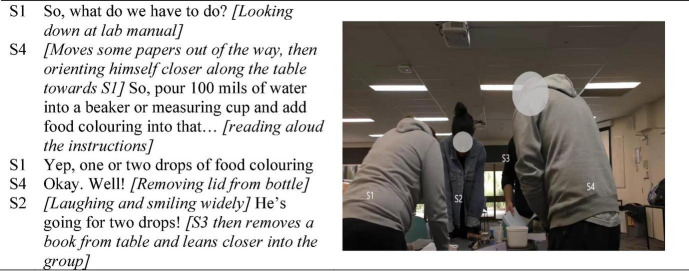
Jointly manifesting positive task-focused interactivity with mild humor in early group life.

In the two illustrative figures, participants are labeled S1 (student 1), S2, etc., according to their seating positions from left to right. Figure 1 is extracted from the opening 5 min of the first activity. Of note is how the group remained standing to undertake the activities after they had together collected their materials and prepared their workspace. Their matching bodily positions shown in Figure 1 (all standing, leaning into the activity) reflects their physical orientation to their activity, and one another (Richmond et al., 2012).

In addition to their physical orientation, S4’s voice-tone carried humored enthusiasm toward their activities. For example, as he removed the cap from the food coloring bottle upon S1 reading aloud “one to two drops,” discovering the bottle had no dropper, his one word “*Well!*” conveyed the situation as humorous. In the voice modulation of this one spoken word there is an orienter-orientee process by which the amusement of the situation—how could the measure of a drop possibly be performed from a wide necked bottle with no dropper—was conveyed (rather than, for example, impatience or mild irritation, such as the utterance of a “*tssk*” sound). S2, making eye-contact, immediately orients to the humor briefly relayed by responding likewise: Her comment and the amusement suggested in her animated voice-tone, matching S4’s humor. Mühlhoff (2015) characterizes the notion of a group’s affective quality as cocreated, by the term *affective resonance*. The brief interaction depicts the ubiquitous enactment of orientation in social situations, in this case facilitating the manifestation of positive affective resonance. The task-focused orienter-orientee coupling was a process of participatory enactment cocreating the social situation as positive in what De Jaegher and Di Paolo (2007, p. 498) described as “the purposeful modulation of the sense-making” of the moment given that S2 responded, in like manner, with brief task-focused humor.

**FIGURE 2:**
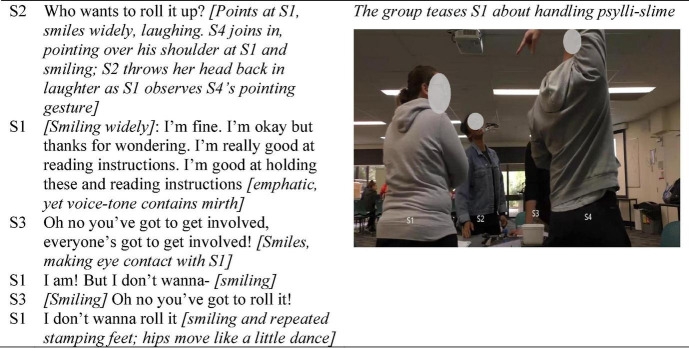
The affordance of humor for relational development and group-level inclusive interactivity.

The next example (Figure 2) demonstrates how the fleeting, mildly nuanced humor referencing task materials, that had been initiated by S4 as they commenced (Figure 1), evolved to incorporate a more *relational* element, which was then tapped as a means for framing the task activities as “all-in” collaboration, serving the purpose of both task and social unity. The intersection of affect and cohesiveness is illustrated as the group utilizes the affordance of spontaneous humored teasing initiated by S2, to achieve coordinated all-group task participation as the joking swiftly evolves into collective bantering. The bantering constitutes their task as co-participatory in all activities rather than just selectively. Affordances of the task itself for cocreating unity is highlighted. The episode is 6 min into the first activity (making psylli-slime) as S1 (laughingly) conveyed her reluctance to handle the materials.

Figure 2 captures the jointly animated, essentially intercorporeal enactment of humor (i.e., S4 pointing elaborately over his shoulder; S2 throwing her head back in laughter and smiling widely; S1 stamping her feet while smiling; S3 emphatically repeating the need to be involved while smilingly signaling humor), which evolved through the lab. For example, 10 min later S1, removing her hand from mixing the oobleck (second activity), extended it dripping, over the container. S4 glanced down at her hand, smiling. S1 made eye-contact with the others, and in a laughter-filled high-pitch tone, exclaimed: “I’ve got into it, now be happy with that!” to which S4, laughs and responds, nodding “that was good participation…” In these fleeting interactions the finely time-scaled orientation process is also evident: S1 held her dripping hand over the container in a manner emphasizing its messiness while gazing from S4 to S3 with communicative expectancy that brought glances toward her hand, to which they responded with smiles.

The episode of teasing about handling the messy materials in Figure 2 is a readily discernible reflection of Fuchs (2016) notion of *embodied interaffectivity* that occurred early in group formation. Interaffectivity was also evident in more subtle, finer-grained actions requiring close attentiveness, such as those which reflect continual orientation in brief gestures (holding dripping hand over container; wry responsive smiles). Figure 2 highlights embodied interaffectivity in the form of humor that although task-focused is also highly relational (i.e., good-humored teasing) through which the group’s idea of amicable collaboration is established, highlighting the deeply intertwined nature of social, and task cohesion as unfolding processes in real time. The brief segment reflects Fuchs (2016) idea that embodied interaffectivity holds the potential for interpersonal understanding, as the participants jointly used task-focused humor, which developed a more relational (i.e., social) flavor in the first steps of developing social understanding of their groupwork as positive (i.e., humorous, friendly, inclusive task interactivity).

#### Interpersonal Attentiveness and Affective Orientation

Attentive awareness of one another was discernible in various forms, such as bodily positioning, actions in handing task materials, and other small moves that innately incorporated interactants in both the conversational realm, and actions that extended physically into their work area, which effectively cocreated what Krueger (2011) calls the “we-space.”

**FIGURE 3:**
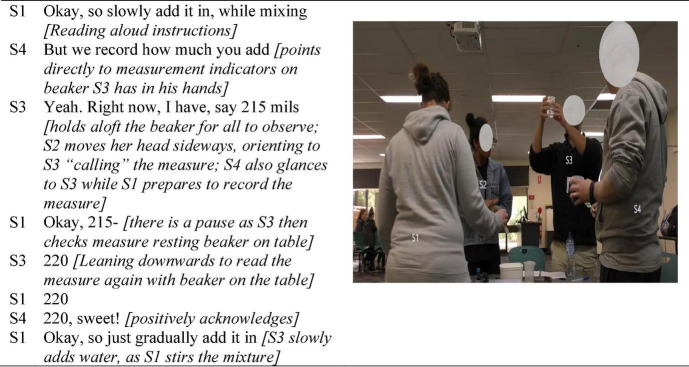
Attentiveness as an interaffective, multimodal process involving bodily, spatial, task, and other artifacts.

**FIGURE 4:**
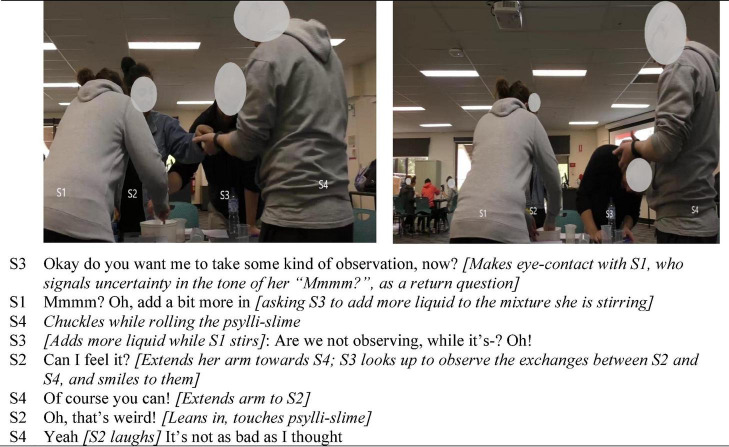
Highlighting group-level corporeal orientation and consistently enacted attentiveness.

The two illustrations presented in Figures 3 and 4 make visible how seemingly inconsequential microlevel actions, taken together and enacted consistently, constituted positive interaffectivity in the ontological flow of interaction, and shaped the tasks as routinely group-level operations. While Bakhtiar and Hadwin’s (2020) research in collaborative learning conceptualized attentiveness to one another as positive socioemotional interaction, the embodiment analysis undertaken here highlighted its important role in group orientation processes. The key role of attentiveness was interpersonal in nature (i.e., not only attention to the task but to one another) expressed through myriad fleeting multimodal actions. The embodiment lens revealed the way in which the material domain also provided affordances for orienting the group, coordinating not only task action but also their affective orientation in transitioning from being relative strangers, to collaborators. For example, members continually held up artifacts so that everyone could get the same perspective of them, as shown in Figure 3, a small but innately inclusive action.

Figure 3 highlights the kind of multimodal microprocesses that ontologically wove together moment-to-moment collaborative action through positive interaffectivity in the form of mutual attentiveness, evident in actions that are explicitly collaborative, enabling joint task understanding in the moment. For example, in the first line although S1 had the task literally in hand she was not herself adding liquid but rather read from the lab book that it needed to be added slowly, to be undertaken by other group hands. S4 added that the liquid needed to be measured which he did both by using the “we” pronoun, and pointing out the measures on the beaker, which combination conveys his utterance in a way that is oriented more *collective* than *directive*. Here he also showed that he did not expect S1 to do all of the instruction reading and interpreting, as he contributed to that process. Although S3 was the person who measured, and poured the liquid, he too enacted this brief activity as a joint process by holding it aloft, “announcing” the measurement, and then sharing that he would now double-check his measurement. In the second-last contribution S4 signaled his acknowledgment not only repeating S3’s call but also adding the positive resonation “sweet.” Taken together, these fleeting actions signal interactive attentiveness enacted multimodally, enabling all-group communication and ongoing orientation, to one another’s actions and to their collective activities.

**FIGURE 5:**
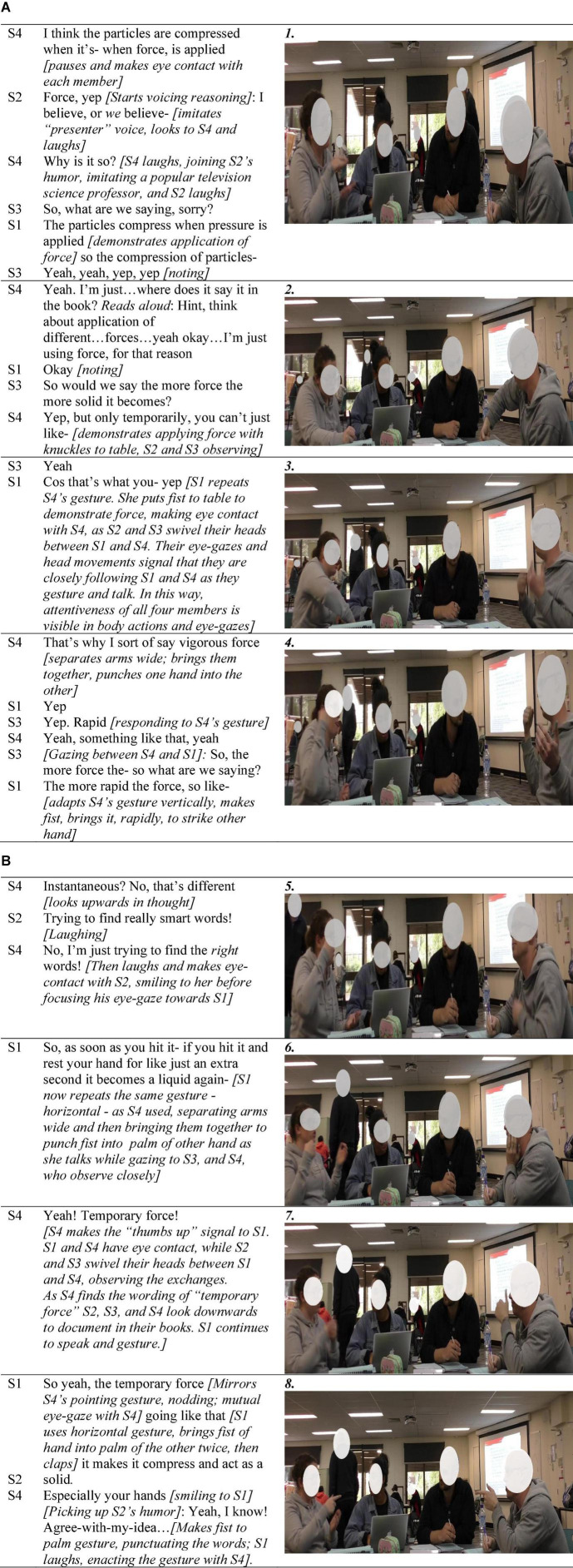
**(A)** Collaborative gestures and group-level attentiveness characterize group task cohesion. **(B)** Collaborative gestures, group-level attentiveness, and task cohesion.

Further, intercorporeality (i.e., as depicted in Figures 1–4) inherently contains a perception-action loop between interactants in which perceiving other/s action holds the potential for prompting like action (Tanaka, 2015; Gallagher, 2017). Figure 4 highlights the role of intercorporeality that appears to be both achieved through, and sustains their attentiveness to one another in coordinating joint task-focused action. Particularly striking was the way the group stood huddled together, relative to the other groups in the class, which typically sat separated by their worktable with one person standing from time to time to undertake a specific task function. In the second picture in Figure 4, for example, another group is visible seated in the background of the picture, and in several of the figures other groups in the class are evident in the background, sitting separated by their worktables (i.e., see also Figures 2, 3, 5).

Figure 4 is taken from 12 min into their first lab together after they have mixed the psylli-slime. S1 was mixing the oobleck, assisted by S3, while S4 rolled out the psylli-slime after it had set. This episode demonstrates how standing together afforded closer physical proximity to one another, and the task, which in turn enabled concurrent progression of the two products in a way that all members could follow the progress of both activities, retaining all-group communication rather than deliberately, or naturally evolving into separate entities (i.e., pairs) to prepare the two products. In this way they achieved, early in group formation, to seamlessly blend concurrently the psylli-slime and the oobleck activities coordinated jointly at group-level, thus maintaining a continual flow of group-level (i.e., cohesive) communication. For example, in Figure 4, S3 offered to document some observations but instead S1 included him in preparing the oobleck. Meanwhile S4 rolled the psylli-slime having removed it from its container once set. His chuckle drew S2’s interest, which elicited an enthusiastic response from S4 (“of course you can!”) and a smile of acknowledgment from S3 while assisting S1.

The way in which this group remained standing during their first activities together enabled animated moving toward one another and their group task—moving toward being a positive attractor state in the most fundamental sense (Sheets-Johnstone, 2009)—the participants implicitly signaled willingness in their readiness for collaborative action. In addition to the interactive flow that was maintained, their close physical orientation around the task may also be viewed as an implicit indicator of attraction to the group and its activities. Thus, the participants’ intercorporeality, in the form of embodied positive interaffectivity, aided coordinated joint action and moreover, signaled their willingness for active co-participation.

Space was also evident in group orientation processes not only in their corporeal proximity but also in continual small movements of making space for one another around, and on the worktable, collectively preparing and clearing, *attending* to the workspace together. This is consistent with Krueger (2011) who posits that the actions through which space is constituted inform interpersonal understanding as “social cognition is fundamentally an interactive form of space management of we-space” (p. 643). Corporeal orientation processes occurring early in group life can signal inclusiveness or alternatively, be manifestly non-inclusive (Richmond et al., 2012). Also, although not the focus of this study, as non-science background students their precision in following and articulating instructions together and documenting measures also indicates early orientation to the practice of scientific experimentation.

Overall, orienter-orientee’s actions within the group were evident from the outset as a multimodal, ongoing micro-temporal process, which can be difficult to observe (De Jaegher and Di Paolo, 2007) given its continual enactment in fleeting actions, and often hidden in plain sight nature.

### (Re)orientation Processes Sustaining Group Cohesion and Positive Group Task Engagement

Throughout their activities, the group continuously engaged in re-orientation processes, physically, materially, spatially, relationally, cognitively, *affectively*, in this way sustaining their cohesiveness and productive, positive group engagement. Ongoing group orientation was evident as they moved between different activities over the course of a lab, and also when the group met to undertake tasks in subsequent labs over the semester. The following two sections illustrate the group perceptually reorienting to new activities (Stevanovic and Monzoni, 2016). The first section captures this process in the group’s interpersonal attentiveness which critically, sustained their cohesiveness during joint science reasoning, and in the second section, reorienting in their next lab together the following week, which facilitated coordinated positive group engagement.

#### Interpersonal Attentiveness During Joint Science Reasoning Sustains Group Cohesion

Given the importance of joint conceptual meaning-making for collaborative science learning, of interest was how the rapport that appeared to develop during the group’s early interactions involving their experimental activities shaped ongoing interaction in the second part of their group task, which was their conceptual reasoning. A fine-grained embodiment lens revealed the critical role of embodied interaffectivity, particularly in the form of attentiveness to one another in sustaining an all-group intellectual space and group task cohesion.

The brief episode, illustrated in Figures 5A,B, is taken from the group’s conceptual reasoning together, which followed experimentation with the oobleck and psylli-slime products, after they had cleaned up and prepared their worktable for their science reasoning. The episode illustrates their interpersonal attentiveness as visibly enacted in the use of collaborative gestures and active listening during the group’s first conceptual reasoning exercise. It highlights the communicative function of attentiveness expressed in bodily actions. Also evident in the group’s joint science reasoning is the carefully modulated orientation of humor, balanced in a way that retains their conceptual focus (i.e., collective intellectual space) until the issue at hand is resolved.

The episode started with S4 sharing his idea, incorporating each member in his gaze. S2 initiated light humor, imitating “presenter” voice, to which S4 responded: “why is it so?” Their brief enactment of a popular television scientist conveys the inquiring nature of the scientist, their joint humor positioning the group as scientists, in a light-hearted manner suggesting their efficacy for the task at hand.

The first picture (Figure 5A) shows S1 gesturing to illustrate the application of pressure compressing particles and is thus a collaborative gesture communicating meaning (Koschmann and LeBaron, 2002). In the second picture, S4 adapted S1’s gesture, extending and refining their thinking following S3’s question. S1 then repeated S4’s gesture (third picture) signaling attentiveness (Koschmann and LeBaron, 2002). S1 and S4 were closely following and reacting to one another’s gestures as S2 and S3 closely observed. The fourth picture shows S4 introducing a new gesture to illustrate “vigorous force,” to which S3 reacted with “rapid.” S4’s response “…something like that” suggests the issue is yet unresolved. S1 then again made a similar gesture to S4 but adapted it vertically, incorporating S4 and S3’s contributions. Their gestural interpretation and adaptation were only possible through their close attentiveness to one another.

In the fifth picture (Figure 5B), S4’s careful orientation of the group was evident when S2 joked about him trying to find “really smart words.” He modulated the perception, trying to find “the *right* words” then briefly laughing with direct eye-gaze to S2, acknowledging her joke, potentially a small action in sustaining relational harmony but maintaining task focus. Further, (see seventh picture), S4 signaled a “thumbs up” gesture, a positive affirmation in the sociocultural context, and in the final image, S1 and S4 jointly point, indicating mutual awareness (Norris, 2004). S2 then jokes to S1 (“especially your hands”). S4 joins in the humor initiated by S2 as he adapts their collaborative gesturing one more time, this time social humor, signifying the end of the episode. In this way, the brief all-group task-focused concentration is eased with joking and laughter, demonstrating the way in which gesturing had both a task and social (i.e., relational) function.

Research in collaborative learning contexts (e.g., Koschmann and LeBaron, 2002; Wardak, 2017; Walkington et al., 2019) has stressed how gestures can have an important role in expressing ideas for which students may not yet have the language to articulate while formulating their thinking, and in signaling joint attentiveness, which was visible in the participants’ body actions. Krueger (2011) explains the use of gestures as “an active structuring and manipulation of we-space—a jointly constituted interaction that serves as a mechanism for driving interpersonal understanding” (p. 649). Through mutual eye-contact and their heads swiveling between S1 and S4, S2, and S3 appeared to be continually active co-participants in cocreating the joint reasoning “we-space.” Their eye-gazing, head movements, and facial expressions illustrate group-level attentiveness during science reasoning. Key here is the continually visible nature of attentiveness in various forms, which, with the careful modulation of light task-based humor in the brief (90-s) segment of their science reasoning, importantly, sustained all-group focus and positive atmosphere.

#### Coordinated Positive Group Engagement

Taking a more distal perspective of the group made visible the participants’ physical orientation in unfolding interactivity over the course of their second lab, also highlighting group-level interaction pattern emergence. A “bird’s-eye” embodied view showed that group (re)orientation appeared to be (informally) led by one member’s (S4) highly animated task-focused actions and continual humor, both of which the other participants oriented to, characterizing the perception-action loop of intercorporeality that Tanaka (2015) describes in his theory of social cognition.

**FIGURE 6:**
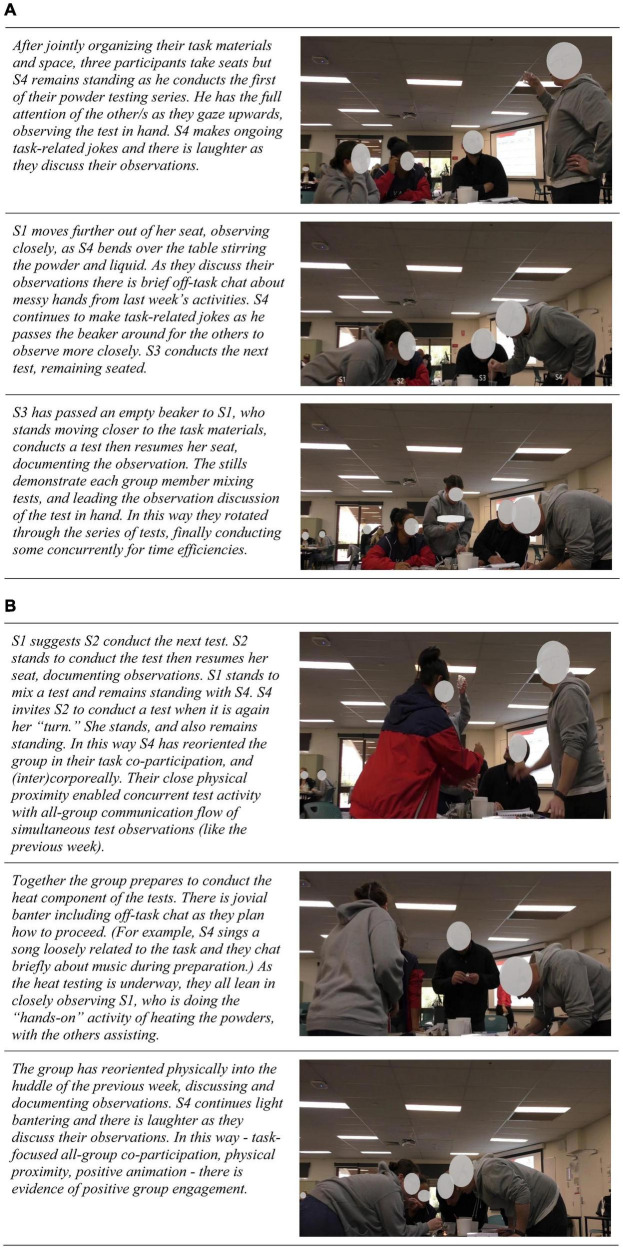
**(A)** Corporeal reorientation unfolding in joint task focus in their second lab together. **(B)** Corporeal reorientation and joint task focus in the second lab.

The illustration of coordinated positive engagement shown in Figures 6A,B comes from the second lab, one week after their first lab together. It involved learning how to make and record observations, identify chemical substances by their unique properties, and the science behind physical and chemical changes through conducting a series of tests mixing four uncategorized (household) powders, with water, vinegar, and then heat. Following experimentation, the group had to document their predictions of the identification of each powder based on their observations. Noteworthy, three group members took their seats to start working while S4 remained standing, but eventually all four were again standing together around their activity.

Overall, Figures 6A,B illustrate how the group-level coordinated positive engagement in their task activities that had emerged in their first lab was maintained in the second lab, as the group reoriented physically by moving out of their seats to stand around their mutual task focus, in bodily expressed engagement with the group and activities. Their highly animated task focus was imbued with a positive atmosphere that appeared to both arise from, and be reinforced by their mutual attentional focus (Metiu and Rothbard, 2013) in a process that makes visible the intertwined nature of affect and cognition, and their task and social cohesion.

Taken together, Figures 5 and 6, reveal how reorientation processes sustained productive group cohesion, and positive group engagement. The embodiment lens made visible the fine-grained temporality of embodied interaffectivity not only in overt enacted behaviors such as joking and laughter but also microforms of action that signaled ongoing group-level interpersonal attentiveness. This was evident in multimodal actions involving talk, gesture, corporeal orientation toward speaker/s, eye-contact, vocal modulation, spatial configuration, task, and other material artifacts in their environment.

### Breakdown and Repair of Positive Interaffectivity to Maintain Group Cohesion

The illustrations above largely focused on how group cohesion *emerged* through the fundamentally affective, interpersonal microprocesses of social interaction that Fuchs (2016) described as embodied interaffectivity. Also, of interest is how breakdown of positive interaffectivity can present a threat to group cohesion but is *maintained* through repair. The kinds of breakdown and repair that permeate social interaction (De Jaegher and Di Paolo, 2007) were observed all through the semester.

In the case group analysis, two perceptible potential threats to the cohesiveness of the group’s on-task interaction were identified: use of a mobile phone for personal, non-group purpose; and positivity peaks with the potential to derail the group task focus. They involved ongoing breakdowns and repairs of interaction in maintaining group cohesion. Two such demonstrations of breakdown and repair are presented below.

#### Use of a Mobile Phone for Personal, Non-group Purpose

Although in the first two labs, two members used their phones briefly to share pictures regarding personal interests (e.g., pictures of cookery), mobile phone use for anything other than a collective purpose by the other members was extremely rare over the semester. However, one student’s personal use of her mobile phone in the first lab (not for group purposes like looking something up or timing experiments, or off-task relational sharing of photos, etc.) was noticeable. Specifically, S2 was observed reading, scrolling, or tapping into her phone in seven instances, which comprised 9.6% of the group task time. When tapping into the phone all of her attentional focus appeared to be directed there, shoulders hunched over the small device, face and gaze directed downwards, no facial or bodily animation toward the group. This reduced the time that the whole group had full task co-participation, and in this way can be interpreted as disrupting task cohesion.

Yet, a fine-grained, embodied analysis of these instances revealed that while she was on her phone, S2 was also simultaneously trying to maintain social contact with the others, by glancing up to make eye-contact and offering occasional small comments. This happened, for example, when humor was being expressed in relation to the task or in off-task small talk. S2 was observed looking up to the speaker, smiling or laughing in response. In this way S2 was making small repair moves in order to maintain social contact with the group.

Later in the semester S2’s mobile phone use increased, more regularly inhibiting all-group task engagement. Remarkably, the video data does not reveal any reactions from the others regarding S2’s repeated phone use, and although there appeared small leakages of irritation from one member in the final lab relating to overreliance of some members for task information, it seemed to be carefully self-regulated (i.e., followed by humor). Therefore, it remains inconclusive whether or not other/s were concerned by S2’s regular scrolling, reading, and tapping into her phone. Personal use of mobile phones during social interaction was found by Aagaard (2016) to create *absent presence* that is negative for the social interaction at hand. In the present study, there was no evidence of a negative impact on group interaction but rather evidence that the harmonious climate was maintained.

#### Positivity Peak With the Potential to Derail the Group Task Focus

In the final lab, there was an atmosphere of fun present, and the group appeared to be in high spirits, all smiling and laughing as they commenced their first task involving predicting and testing the buoyancy of different size and density objects in a tub of water on their worktable. This positivity peak may have derailed the group task focus and cohesiveness, but subtle repair illustrates the group’s ongoing effort to maintain positive interaffectivity and cohesion.

For example, the following brief interactions occurred when S2 playfully threw a ping-pong ball (a test object) at S1’s face early in the lab:

S2 Hey! [seeks S1’s attention, throws ping-pong ball at S1, hitting S1’s glasses. S2 is highly animated, laughing loudly, glancing briefly toward S4 who continues writing. S3 glances toward S2 smiling and continues interacting regarding the task].

S1 These are expensive don’t throw stuff at them! [*S1’s face registers surprise and she smiles].*

S3 [*In the meantime, S3 and S4 continue on-task]*: That’s a golf ball or something, that one *[refers to a ball he has picked up which has outer casing removed].*

S4 I think golf balls float too *[responding to S3].*

S3 *[Responding to S4]* Yeah.

S2 It actually didn’t look like it hit you—it looked like it just came here and then *[gestures with her arms indicating the ball ricocheted off S1’s glasses. S2 appears to be initiating repair work as she leans her torso and head down to look into S1’s face for direct eye-contact, or possibly checking S1’s glasses. S4 glances up at S2, then returns to his documenting].*

S1 It gave me a bloody fright! *[S1’s voice tone sounds cheerful but also emphatic as she starts to write in her book].*

S2 Aaahhhh *[Still smiling, S2 returns on-task, writing].*

As the ball hit S1’s glasses, S2 laughed and glanced to S4 but S4 was preparing to record their observations and had one elbow resting on the worktable, with hand extended over his eyebrows in a pose of concentration. S1 twice commented in a friendly positive manner suggesting S2 desisted the play, first remarking the glasses are expensive and then in response to S2’s initiation of repair, that it gave her a fright, in turn repairing S2’s understanding since S2 was still laughing animatedly. S1 did this in a way that retained the positive group tone as she smilingly told S2 not to throw things at her glasses. S1 then looked down and started writing in her book. The atmosphere remained jovial, however, S2’s evident peak of excitement quietened as the others continued on-task. The non-joining of S3 and S4 in S2’s play could be viewed as not further encouraging it and thus subtle repair work, orienting S2 back on-task. Stevanovic and Monzoni (2016) similarly found that repair of social interaction can be verbal or unspoken actions, and it is the actions that actors typically orient to, which in our case study, it is the continuation of task-focused activity rather than play, that the other participants enact and S2 therefore subsequently orients to.

De Jaegher and Di Paolo (2007) posit that such breakdowns and repairs are frequent, and that this perspective of social interaction and its coordination, through the microprocesses of co-orientation, breakdown, and repair “allows us to connect interaction dynamics with sense-making” (p. 496). The brief exemplar above illustrates, as De Jaegher and Di Paolo (2007) put it: “How the individual sense-making [is] adjusted through the situation…in this case created by the interaction dynamics and not just the individuals” (p. 498). The brief episode reflects findings of other collaborative learning studies (e.g., Tomas et al., 2016; Costa et al., 2017; Volet et al., 2019) that have observed the need for managing not only negative, but also positive affect during groupwork so that it does not spiral, distracting the group’s task focus. An embodiment lens made visible the microprocesses of breakdown and repair actions that enabled the group to achieve this through their group dynamics.

Finally, also evident in the group’s final lab together, was their highly animated interactivity which included humor and laughter, with ongoing processes of positive affective orientation involving its modulation for task focus at a micro-sequential level, as it emerged early in this group’s life, which suggests macro-temporal patterns of embodied positive interaffectivity and cohesiveness over the semester.

## Discussion

Group cohesion was explored as the dynamically emergent positive interaffectivity of four first year university students who reported an enjoyable and cooperative experience at the end of their semester of science groupwork. The illustrative case analysis presented above was inspired by Ferreira’s (2021) argument that embodied perspectives may offer new insights and avenues for collaborative learning research. In this paper we have integrated literature from anthropology, communication, philosophy, psychology, and sociology, which together provide valuable conceptual insights regarding the microprocess of interpersonal affect as embodied interaffectivity in social interaction, making possible their exploration in group cohesion.

A fine-grained embodied analysis made visible the interplay of key microprocesses that have been conceptualized in cross-discipline theoretical perspectives, and their evolution over time as macro-temporal patterns, magnifying their visibility and collective impact. Critically, since most group cohesion research relies on aggregated *post hoc* self-reports, our analysis showed what the emergence and development of positive embodied cohesiveness looks like.

A holistic embodiment lens enabled a fully situated picture, integrating participants’ corporeal presence in combination with use of space, task, and other material artifacts, thus making visible their systemic interplay in a flow of relationality which together cocreated the group’s positive collaborative affordances through their spontaneous actions. An embodied perspective illuminated the multimodal microscale actions that achieved the group’s positive interaffectivity and importantly, revealed that developing, and maintaining cohesion is an act requiring whole-group effort.

This holistic analytical approach unveiled the social and dynamic nature of affect—its inherently interpersonal quality—to be an innately multimodal phenomenon. It also brought to light the environmental affordances (Santuber et al., 2020) of the situation for productive collaboration, and most importantly the extent of their uptake by the participants. The uptake of collaborative affordances warrants further research, for example understanding who, when, how, and to what extent are environmental affordances capitalized, and situational constraints overcome. Interactions between the development of interpersonal affect in groups and cognitive load demands of tasks and contexts also remain an area of future systematic exploration. In focusing on a fine-grained embodied analysis of interpersonal affect as a lens to examine the emergence and maintenance of group cohesion, this work opens up rich opportunities to further examine the complex ecology of groupwork. The main findings of this study are considered in the following sections, in terms of their insights into effective collaboration, and the potential for further research in these areas.

### The Key Role of Orientation and Interactional Antecedents of Positive Interpersonal Affect

The process of orienting from their first moments together appeared vital to the co-construction of positive interaffectivity, reflecting Goodwin’s (2000) observation that coordinating social interaction is a process of visible co-orientation. In this way, the interactional antecedents of positive interpersonal affect that facilitated the group’s cohesiveness emerged during their first lab together as participants oriented to one another physically (i.e., corporeally, spatially, materially) and relationally through their task interactivity using light humor. Orienting as a group was a highly intercorporeal process, and as the illustrations showed, in this way they stood out from the rest of the class, standing to conduct their first experimental activities together. Goodwin (2000) argues that such “displays of postural orientation…build participation frameworks” that “help establish the interactive ground” (p. 1519). Our analysis also revealed the important role of intercorporeality and interaffectivity (Fuchs, 2016), the way in which, when we are in social interaction our corporeality is a systemic component.

The analysis provides an empirical account of the microprocesses of orienting in social interaction described in De Jaegher and Di Paolo’s (2007) embodied theory of social cognition as participatory sense making, and how they evolved in cocreating the group we-space as positive and cohesive over time. Our analysis of the microprocesses of orienting thus extends emotion scholars’ Van Kleef et al.’s (2010) emotions as social information theory (EASI) by operationalizing an explicitly embodied account of how participants’ affectivity provided information about “their inclusionary status in the group, the norms of the group, and the functioning of the group as a whole” (Van Kleef et al., 2017, p. 159).

The group’s animated vibrancy—a striking feature of their first two labs together—appeared to be afforded in part by their standing positions which facilitated closer physical proximity, securing collective focus, and making task artifacts jointly, readily accessible. While sounding routine, task artifacts are often used in groupwork in ways that position some as empowered while marginalizing other/s (Wieselmann et al., 2021), even unintentionally. Instead, the group’s spontaneously unfolding actions involved ongoing efforts to adapt to environmental constraints (Haupt, 2015) such as overcoming being cut off from one another and the activity at hand by the confines of the worktable, even stirring mixtures collectively, enabling a truly collaborative effort and collective focus. Our analysis found that the group maximized the environmental affordances of their intercorporeality (i.e., in-person presence together) to cocreate a positively charged collaborative we-space around the activity materials. More case study research is needed to examine how the body is used in orienting as purposefully positively interactive, which is underexamined in collaborative learning, and as Ferreira (2021) noted, the extent to which participants’ use their bodies in ways that promote or impede collaboration. Given the highly influential role that interpersonal affect in early group life has for ongoing interaction (Barsade and Knight, 2015), systematic research on the microprocesses of affectively orienting as a group will provide more insight on this important group development phase.

### The Role of Humor in Group Cohesion

The group’s co-orientation to mild task-focussed humor commenced in a socially safe manner and evolved over time into more socially focused humor, revealing that the task itself was the vehicle through which they could build rapport and get to know one another, the affordance offered by the task for relational development. Our findings showed that task and relational humor were antecedents of the group’s cohesion, which corroborates other research revealing the positive role humor can play in collaborative groupwork. For example, Lobczowski et al.’s (2021) research with graduate students which showed humor was a socioemotional regulation strategy, and in workplace teams, Lehmann-Willenbrock and Allen (2014) found group-level humor patterns positively related to performance. Our finding that humor needed to be carefully modulated to maintain task cohesion also aligns with other studies that found high positivity could override task focus, at different levels of collaborative learning, from school (Tomas et al., 2016), to undergraduate (Volet et al., 2019) and postgraduate (Costa et al., 2017; Lobczowski et al., 2021) contexts. Our illustrative analysis extends the extant research by unveiling how humor emerged, was enabled (co-orientation) and sustained by participants as evolving microprocesses. For example, how it initially emerged in co-orientating to humor (i.e., reciprocal) through task materials, its evolution to more social (i.e., relational) interactions, and importantly, how it was modulated in a way that maintained group harmony, and breakdown and repair moves that tempered threats to group cohesion. To date, the role of humor has been underexamined in groupwork contexts. A better understanding of how humor functions in diverse sociocultural contexts, its negative-valence forms such as sarcasm, ways that may marginalize, and how it is modulated, and by whom, is crucial for educators and managers in multicultural educational and workplace contexts, calling for further research in this area.

### The Significance of Reorienting to Positive Interaffectivity in Subsequent Activities

Evidence of group members’ building on their early positivity and task cohesion as they perceptually (re)oriented to new activities (Stevanovic and Monzoni, 2016) was visible in participants bodily expressed interpersonal and task engagement and the positive atmosphere that accompanied their mutual attentional task focus. The group’s process of positive task engagement illustrated the deeply intertwined nature of affect and cognition, and task and social cohesion, constructs which although often separated for analytical purposes, ontologically unfolded as entwined.

The kind of physical proximity around task artifacts, signaling joint attentional focus, combined with positive interaffectivity as displayed by our case group has also been found to reflect positive group engagement among high functioning professionals in MBA teamwork (Costa et al., 2017) and in the workplace (Metiu and Rothbard, 2013). In their longitudinal ethnographic study of contrasting technology projects, Metiu and Rothbard (2013) identified that the shared positive affect of the successful team appeared to both result from, and strengthen joint attentional focus. Fuchs (2016, p. 196) posited that embodied interaffectivity can generate “self-sustaining interaction patterns that go beyond the behavioral dispositions of isolated individuals” such that affect can be conceived as part of the “intercorporeal space” of the group. This also aligns with sociologist Collins’ (2004) interaction ritual theory through which Metiu and Rothbard (2013) interpreted their successful project group’s interactions. Interaction ritual is described by Collins (2004) as “mutually focused emotion and attention producing a momentarily shared reality, which thereby generates solidarity and symbols of group membership” (p. 7). In this way, Collins (2004) argues, joint activity elicits a common focus and a sense of belonging in the moment that can be experienced as positive elevation. This is reflected in the animated vibrancy of the group’s humored interaction that developed during early group life, which through repetition, sustained the intercorporeal space as one of positive interaffectivity.

The present study extends this work by illustrating how a group of first year university students from different backgrounds, in an introductory science unit, managed to achieve the kind of positive group task engagement attributed to the interactive processes of high functioning professionals. The explicit focus on positive interaffectivity as cohesive and productive showed, through examining the microprocesses of reorienting, that it takes place in small actions of which we are all typically capable.

The findings showed how the social information that shapes groups, is conveyed (Van Kleef et al., 2010, 2017) through ongoing microprocesses of (re)orienting in social interaction. The function of orienting in social interaction, in establishing the implicit norms that are pervasive in groupwork, should be explored further given that the “underlying mechanisms” of group norms remains “one of the big unsolved problems” for collaboration (Santuber et al., 2020, p. 17).

### The Key Role of Attentiveness Through Co- and Re-orientation

A vital but less prominent linchpin that appeared to secure the group’s *esprit de corps* in the present study is the way in which attentiveness was enacted explicitly as an interpersonal action in orienting to one another as a group (i.e., not just attentiveness to the task). Specifically, their ongoing practice of *orienting to the task through one another* appeared to be what constituted their interactivity as truly collaborative. This was made visible by phenomenologically following (Goodwin, 2000; Rödel, 2020) the participants’ unfolding simultaneous actions (i.e., where participants’ attention was directed moment-to-moment). Most striking about our case group, is how the group, although not undertaking an activity designed to facilitate embodied collaboration, instead overcame situational and task constraints (Haupt, 2015) to achieve their truly collaborative effort of conducting their science tasks through one another (de Freitas et al., 2019). Importantly, this embodied-driven microanalysis showed how the group members enacted collaborative learning not just with each other but through each other. This is a salient qualitative finding that warrants systematic investigation in different group contexts.

Attentiveness to one another was a subtle but key characteristic in the group’s interactivity that emerged early, facilitated their co-orientation, and reorientation as the group undertook subsequent activities. The collaborative gesturing and close attention exhibited a continuation of participants’ task focus through one another, which was made possible due to their sustained interpersonal attentiveness. Although not the focus of our paper, the richness of conceptual discussion the group achieved in their first lab together (briefly illustrated in Figures 5A,B) was built on the rapport established in their initial embodied actions. This highlights the affect-cognition synergy of social interaction, and reflects Ferreira’s (2021, p. 12) observation that “the process of meaning-making thus takes place at a level of interpersonal perception.”

The innately affective nature of attentiveness has been observed in other collaborative learning contexts (e.g., Barron, 2003; Rogat and Linnenbrink-Garcia, 2011; Ucan and Webb, 2015; García et al., 2020). The embodied perspective adopted in the present study highlighted the proliferate nature of microlevel orienter-orientee interactions involved in group orientation and the importance of what de Freitas et al. (2019) call *response-ability*, consistent attentiveness as a form of embodied interaffectivity and antecedent of group cohesion. Moreover, our fine-grained analysis of (re)orienting processes in the group’s interaction illustrated how successful collaborative learning extends beyond shared interactivity and is achieved in continually orienting to their various activities *through one another*.

### Cohesion Is a Consistent Whole Group Effort of Attentiveness

The case group analysis also illustrated how even when not contributing to the group through talk, participants nevertheless made the important contribution of holding the group space as cohesive with communicative signals of attentiveness such as smiles, nodding, and eye-contact. These communicative signals during groupwork appear salient but remain under-researched. In multimodal analysis eye-contact is viewed as interaction (Norris, 2004), which is an important point because more quiet students are often perceived as low or non-contributory. Our analysis provides strong indicators that the consistent attentiveness that was evident in body signals (rather than behaviors such as gazing around the room or other bodily actions or inaction signaling non-attentiveness) are critical in maintaining group-level task-focus and cohesiveness.

Acknowledging interpersonal attentiveness in task interaction as a critical element of all-group participation (i.e., task cohesion) calls for a better understanding of the potential contribution of more reticent participants. This is an important avenue for future research given that groupwork in learning and working contexts increasingly involves socioculturally diverse participants. Our embodied analysis unveiled the important role that less active participants can play in maintaining group cohesion, not only by remaining attentive, but also through their attentiveness being acknowledged (e.g., frequent inclusive eye-contact) by other more active peers. While there is always potential for groups to split either deliberately or naturally, for example along the lines of more active and quieter contributors, this was avoided in this successful group through consistent small actions of interpersonal attentiveness. The extent to which other actions may also play a role should be established through further fine-grained holistic analyses, since as Erickson points out, “during the course of interaction nobody is ever doing nothing. Everyone is doing something in every moment while others are doing what they are doing—constituting together a continuously enacted ecology” (Erickson, 2017, p. 60) thus the systemic nature of group processes in which all are co-contributors to higher level construct emergence.

Self-report measures of group cohesion, which are typical of prior research, cannot capture the microprocesses involved in real time group cohesion. It is through the embodiment lens that a real time systemic and insightful perspective of interaffectivity and group cohesion in terms of who was doing what and when in relation to one another in the flow of interactivity was revealed. Nevertheless, and as illustrated in the present study, combining the close examination of interaffectivity in the emergence and maintenance of group cohesion with participants’ self-reported accounts offers not only valuable insights into this phenomenon, but directions for future research on the impact of such seemingly inconsequential behaviors.

### Conceptual Affordances of Embodied Perspectives for Studying Multimodality in Communication

Finally, taking an embodied perspective to examine our case group illuminated the group’s highly animated, multimodal interaction, which is referred to by Norris (2004) as “high modal density” which, she argues, “equates to a high level of interactional attention/awareness” (p. 94). Such high level attentiveness appeared necessary for the mutual orientation that facilitates joint social understanding. This finding is consistent with De Jaegher and Di Paolo (2007) who posited that a high degree of orienter-orientee interaction that achieves mutual orientation, holds the potential for the kind of truly collective meaning-making that constitutes effective collaborative learning. As Goodwin (2000) argued, such phenomena show that “any participation framework is an ongoing contingent accomplishment…that has to be continuously achieved through public displays of orientation within ongoing processes of interaction” (p. 1500).

The group’s highly animated multimodal communication during their first lab activity made visible the interpersonal attentiveness of participants to external observation, and therefore it can be assumed was also apparent to the participants themselves (Bartel and Saavedra, 2000). Given that we are primed from birth to be keenly aware whether others are paying us attention (Norris, 2004), exhibiting high attentiveness distributed across the group (Stephens and Lyddy, 2016) early in group life appears important since cohesion needs to emerge swiftly in short term groups (Salas et al., 2015) which this group achieved. Furthermore, teamwork research has also consistently shown the important function of early interaction in shaping ongoing group processes (Barsade and Knight, 2015; Braun et al., 2020). Our fine-grained analysis through an embodiment lens further extended the findings regarding the criticality of early group life for ongoing interaction by showing that the high communicative multimodal density (Norris, 2004) of the group may have played a key role. More systematic studies of the multimodality of group interactivity processes at different temporal stages may provide fruitful new insights.

### Participants’ Awareness of Interpersonal Affect and the Role of Individuals Within the Group

While discussing their groupwork at an end of semester group interview, participants reflected on what they perceived as not only their positive relations but also efficiency in conducting their experimental activities together, commenting that: “We kind of had a routine…we didn’t even talk about that but it just kind of happened” (S4). Then, later in the discussion, reflecting on how they coordinated their conceptual reasoning in a way that secured all-group participation, S1 stated that “it just happened.” In light of the richness of the observed evidence of interaffectivity in the emergence and maintenance of group cohesion, the participants’ recollections reflect the unlikelihood that these kinds of fleeting, and seemingly typical routine interactions will be readily recalled or recounted retrospectively as potentially significant (Erickson, 2006; Herrle, 2020). Yet, the participants’ perceptions at semester end represent their accumulated moments of interaction, thus demonstrating the relevance of micro-temporal everyday behaviors that, taken together, ontologically constituted the group’s cohesiveness. Retrospective self-reports remain minimally informative in terms of understanding the forms, and significance of interaffectivity in contributing to the group’s success.

Although the analysis of participants’ accounts focused on the (collective) interactivity, the “we-space,” the individual differences that were visible in the group highlighted the fact that students enter collaborative learning situations with more or less collaborative awareness. For example, and as illustrated through the embodied analysis, the process of (re)orienting as a group in the second week involved highly animated task-focused actions and continual humor, which was clearly led by one member (S4). The informal leadership of S4 from the first lab appeared invaluable and importantly was welcomed by the others, which was evident by the group’s co-orientation. In stark contrast, S2’s personal mobile phone use disrupted task cohesion. Research on mobile phone use during social interaction (e.g., Aagaard, 2016) found that it creates “unintentional misattunement” (p. 8), which can signal disinterest and disrupts the coordination that is facilitated through interactive microprocesses of eye-contact, facial gestures, and timely responsiveness, the corporeal animation that signals attentive presence. This dynamic, Aagaard (2016) argues, is negative for the social interaction at hand. While S2 appeared to stay attuned socially, such as glancing up to join humor and laughter, in this way maintaining social presence, there appeared a lack of collaborative awareness regarding task contribution, which may not be so well tolerated in other contexts.^1^ Our focus on a positive outcome from a newly formed group of disparate learners offers promise that with awareness regarding continually practicing these small things, taken together they can make a difference to affectivity, cohesiveness, and collaborative capacity in groupwork. These findings call for further research into the kinds of participatory roles played by individuals in groupwork (Heinimäki et al., 2021), and for extending this research through an embodied analytical perspective.

## Concluding Comments

Focusing a fine-grained analytical lens on a newly formed group, from different backgrounds in their first year of university, and who achieved a positive cohesive group experience, highlighted the interplay of valuable but underexamined microprocesses that warrant further research attention, and raise questions regarding awareness for adult participants entering collaborative groupwork. Although illustrated in a single case, the findings are highly relevant in light of the wide collaborative variability that continues to be reported in the literature, and reports of reticence among university students for groupwork in the context of twenty-first century skills requirements, and pressing societal needs that are both global and local, for joint social understanding.

In their review of higher education teamwork pedagogy literature, Riebe et al. (2016) report the rather striking finding that the agency of the students themselves in achieving their collaborative outcomes has largely been ignored. Our embodiment lens illuminated the kinds of taken for granted actions that taken together, increased collaborative affordances. These were small, everyday behaviors that with awareness, can be practiced in higher education to enhance collaboration skills and collaborative outcomes.

An embodied perspective of the function of interpersonal affect in group cohesion found that cohesiveness was an all group effort, of which task and relational humor were antecedents, along with the important role of interpersonal attentiveness that facilitated the participants’ undertaking their learning activities through one another, constituting their learning as truly collaborative. By making visible how positive interaffectivity and cohesiveness emerge and are sustained over time in the kinds of common microprocesses of interaction that coordinate a group’s interactivity and facilitate their collective social understanding, the embodied lens represents a promising conceptual perspective for future research in this field.

## Data Availability Statement

The datasets presented in this article are not readily available because participants did not consent to open access to this data at the time of data collection and did not give consent for this data to be available to other researchers not affiliated with the original research team. Therefore, access to the datasets cannot be provided. Questions regarding the datasets should be directed to CJ at c.a.jones@murdoch.edu.au.

## Ethics Statement

The research was approved by the Murdoch University Human Research Ethics Committee. The participants provided their written informed consent to participate in the research. Written informed consent was obtained from all participants for the publication of any potentially identifiable images or data included in this article.

## Author Contributions

CJ conceptualized the study, undertook the data analysis, and drafted the manuscript. DP-P and SV validated the data analysis. All authors contributed to the manuscript revision, read, and approved the submitted version.

## Conflict of Interest

The authors declare that the research was conducted in the absence of any commercial or financial relationships that could be construed as a potential conflict of interest.

## Publisher’s Note

All claims expressed in this article are solely those of the authors and do not necessarily represent those of their affiliated organizations, or those of the publisher, the editors and the reviewers. Any product that may be evaluated in this article, or claim that may be made by its manufacturer, is not guaranteed or endorsed by the publisher.

## Footnotes

1 It is possible the student (S2) who used their mobile phone for personal purposes may have spoken off camera to the group regarding an (ongoing) need for this.
